# Lean Body Mass Associates With a Hypertensive Cardiovascular Phenotype in Men but Not in Women

**DOI:** 10.1002/jcsm.70125

**Published:** 2025-11-10

**Authors:** Dulanji Gunawardana, Meihan Guo, David Montero

**Affiliations:** ^1^ Faculty of Medicine, School of Public Health Hong Kong University Pokfulam Hong Kong; ^2^ Department of Medicine Beth Israel Deaconess Medical Center, Harvard Medical School Boston Massachusetts USA; ^3^ Department of Medicine, School of Clinical Medicine Hong Kong University Pokfulam Hong Kong; ^4^ Libin Cardiovascular Institute of Alberta University of Calgary Calgary Canada

**Keywords:** cardiac remodelling, hypertensive ventricular remodelling, regional lean body mass, sex dimorphism, total lean body mass

## Abstract

**Background:**

Lean body mass (LBM) is independently associated with the function and structure of the cardiovascular (CV) system in women and genetically predisposed men with low LBM. Yet, the relationship between LBM and the CV system remains uncertain in the general population comprising a wide spectrum of LBM.

**Methods:**

A total of 325 healthy women (*n* = 162) and men (*n* = 163) throughout the adult lifespan (18–78 years) matched by age (age = 43 ± 18 vs. 44 ± 18 years) and physical activity were included. Body composition, including LBM, fat and bone mass, was assessed in total and per body region (legs, arms and trunk) by dual‐energy x‐ray absorptiometry. Left ventricular (LV) mass and structure as well as peripheral and central haemodynamics were determined via echocardiography and continuous blood pressure measurements.

**Results:**

Women presented with lower total LBM (*p* < 0.001) and higher body fat (*p* < 0.001) than men. Total LBM did not associate with systolic blood pressure (SBP) in women (*p* = 256) but did positively associate with SBP in men (*r* = 0.31, *p* < 0.001). Total LBM did not associate with LV concentric hypertrophy (LVRWTd) in women (*p* = 448) but did positively associate with LVRWTd in men (*r* = 0.24, *p* = 0.003). Similar sex‐specific associations were observed for regional LBM, except for arm LBM, which associated with all study variables in women. Adjustment by body fat or body fat percentage did not modify the results.

**Conclusions:**

Total LBM independently associates with a hypertensive CV phenotype in men, whereas regional LBM, specifically in the arms, is linked with the same detrimental phenotype in women.

## 1 Introduction

The accretion of muscle mass, particularly in men, is generally linked to aesthetic improvement. Whether high muscle mass associates with physiological benefits and hard clinical outcomes is unclear because previous investigations have mainly focused on the low end of the muscle mass spectrum [1]. Yet, recent pilot studies have disclosed independent associations of lean body mass (LBM), that is, the common surrogate of muscle mass, with cardiac and aerobic capacities, notably in women as well as in genetically predisposed men with low LBM [2, 3, 4]. In these individuals, increased LBM might elicit functional and structural adaptations in the cardiovascular (CV) system strongly linked to lower CV and overall mortality [3, 4]. Specifically, higher LBM positively associates with the reduction of systemic vascular resistance (SVR), augmented venous return and left ventricular (LV) filling, contributing to the optimal ‘eccentric’ myocardial remodeling of the heart [3, 4]. If these findings were applicable to the general population, LBM could be hypothesized to be an effective and wide‐ranging preventive target—unlike other phenotypic variables in the CV system, LBM is readily amenable to modification. The present study assessed the relationship of LBM with peripheral and central haemodynamics, LV mass (LV_mass_) and structure in a representative sample of the general population of adult non‐obese women and men, comprising a wide spectrum of LBM.

## 2 Methods

A total of 325 women (*n* = 162) and men (*n* = 163) throughout the adult lifespan (18–78 years) matched by age (age = 43 ± 18 vs. 44 ± 18 years, *p* = 0.595) and physical activity (total moderate‐to‐vigorous physical activity [MVPA] = 5.3 ± 3.4 vs. 5.9 ± 3.8 h·week^−1^, *p* = 0.090; endurance‐specific MVPA = 4.7 ± 3.3 vs. 5.2 ± 3.8 h·week^−1^, *p* = 0.234) were included. No intentional matching procedure was needed. All individuals were non‐smokers, non‐alcoholic and non‐obese (body mass index < 30 kg·m^−2^). Inclusion criteria comprised healthy status according to health/clinical questionnaires and resting ECG/echocardiography screening, absence of current medical symptoms and medication and no history of chronic disease. The study was approved by the Institutional Review Board of the University of Hong Kong (UW 21‐401/22‐025)/Conjoint Health Research Ethics Board (REB18‐1654) of the University of Calgary and conducted in accordance with the declaration of Helsinki.

The participants were instructed to avoid strenuous exercise, alcohol and caffeine from 24 h and to record fluid intake for 5 h prior to their visit to the laboratory. Before starting the testing protocol, the participants rested in supine position for 15 min on the testing platform in which all measurements were performed. Body composition, including LBM, fat and bone mass in total and per body region (legs, arms and trunk), was determined via dual‐energy x‐ray absorptiometry (DXA) (Hologic QDR 4500) following best practice recommendations [5]. High‐resolution ultrasound (Mindray Medical M9) was used to assess cardiac structure and function at rest. LV stroke volume (SV) was determined as left ventricular end‐diastolic volume (LVEDV) minus LV end‐systolic volume (LVESV), whereas the product of SV and heart rate (HR) provided cardiac output (Q) [3]. Resting arterial blood pressures (systolic blood pressure [SBP], diastolic blood pressure [DBP] and mean arterial pressure [MAP]) were measured via the volume‐clamp method in the middle finger of the left hand, calibrated to brachial artery pressure and positioned at heart height level (CNAP Monitor 500 HD, CNSystems). SVR was defined as the ratio of MAP and Q. The recommended Cube algorithm was used to determine LV_mass_ (LV_mass_ = 0.8 × 1.04 × [(interventricular septum at end‐diastole (IVSd) + LV internal diameter at end‐diastole (LVIDd) + LV posterior wall thickness at end‐diastole (LVPWDd))^3^ − LVIDd^3^] + 0.6) [6]. LV relative wall thickness (LVRWT) was calculated as LVRWT = (2 × LVPWd)/LVIDd. Peak O_2_ consumption was assessed via an incremental cycle ergometry test, as previously described [3]. Statistical analyses were performed with SPSS 26.0. Linear regression analyses were implemented to assess the relationship of total or regional LBM with peripheral/central haemodynamics and LV structure. The Pearson's correlation coefficient (*r*) was determined without or with adjustment by total or regional body fat or body fat percentage.

## 3 Results

General characteristics, cardiac structure and body composition variables are presented separated by sex in Table S1. Cardiorespiratory fitness (VO_2peak_) and blood pressures were in the healthy range according to age and sex. With respect to cardiac structure, LV_mass_ and its components (IVSd, LVIDd and LVPWd) were decreased in women relative to men (*p* < 0.001). LVRWTd, a variable reflecting LV remodeling, did not differ between sexes (*p* = 0.697). Regarding body composition, women had lower LBM (*p* < 0.001) and higher body fat (*p* < 0.001) than men in total and per body region except the trunk.

**FIGURE 1:**
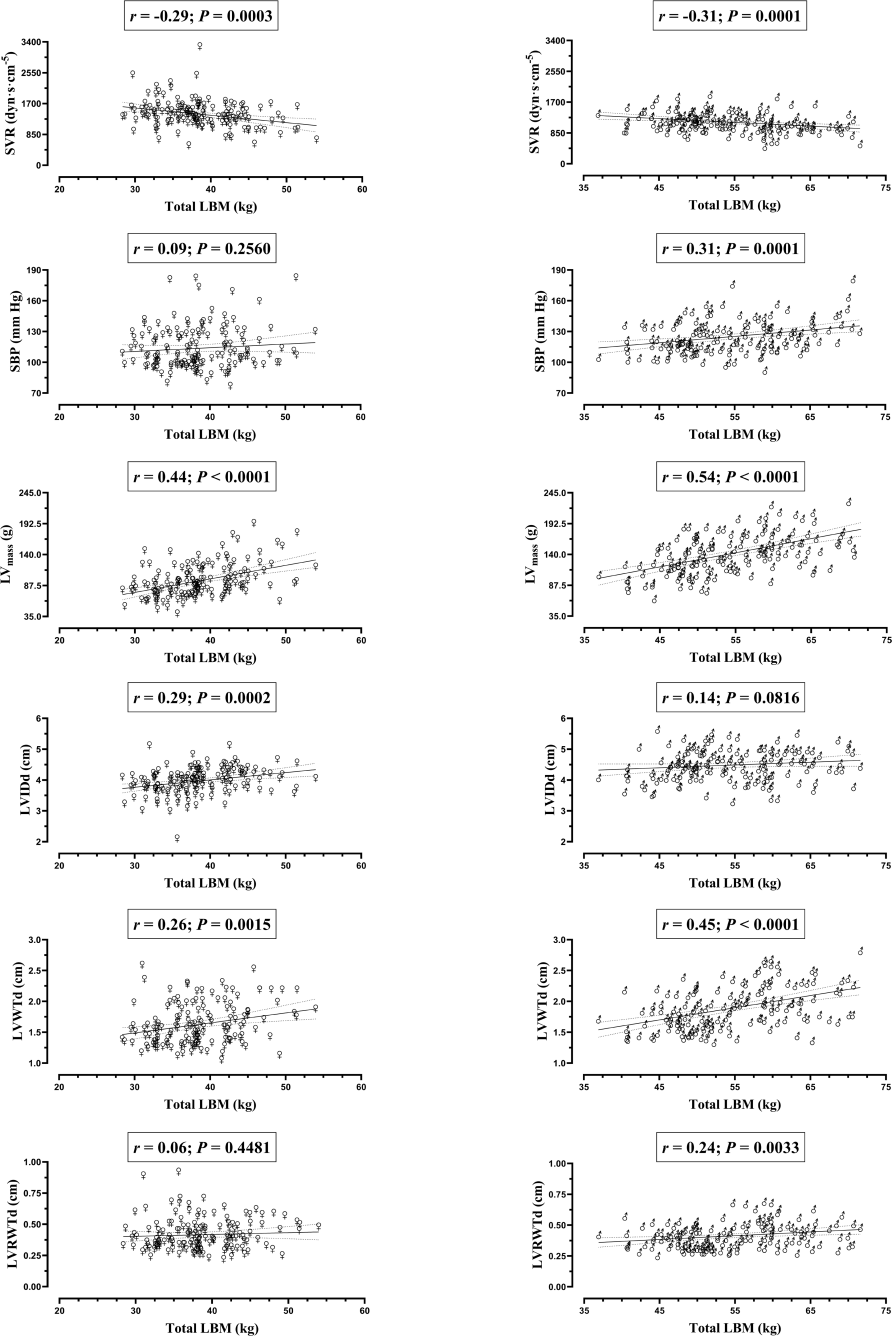
Association of total lean body mass (LBM) with peripheral/central haemodynamics, left ventricular mass (LV_mass_) and structure in women and men. Each graph includes the line of best fit with 95% confidence interval. LVIDd, left ventricular internal dimension at end‐diastole; LVRWTd, left ventricular relative wall thickness at end‐diastole; LVWTd, total left ventricular wall thickness at end‐diastole; *r*, Pearson correlation coefficient; SBP, brachial systolic blood pressure; SVR, systemic vascular resistance.

Figure 1 displays the association of total LBM with peripheral/central haemodynamics and LV structure according to sex. Total LBM inversely associated with SVR in women (*r* = −0.29, *p* < 0.001) and men (*r* = −0.31, *p* < 0.001). In contrast, total LBM did not associate with SBP in women (*p* = 0.256) but did positively associate with SBP in men (*r* = 0.31, *p* < 0.001). Regarding LV structure, total LBM positively associated with LV_mass_ in women (*r* = 0.44, *p* < 0.001) and men (*r* = 0.54, *p* < 0.001). However, total LBM positively associated with the internal size of the LV (LVIDd) in women (*r* = 0.29, *p* < 0.001) but not in men (*p* = 0.082). Moreover, total LBM did not associate with the geometrical index of LV concentric hypertrophy (LVRWTd) in women (*p* = 0.448) but did positively associate with LVRWTd in men (*r* = 0.24, *p* = 0.003). Similar sex‐specific associations were observed for regional LBM, except for arm LBM, which associated with all study variables in women (Table S2). All associations remained significant after adjustment by the family‐wise error rate, except for ‘Arm LBM‐DBP’, ‘Arm LBM‐LVIDd’ and ‘Trunk LBM‐SBP’ in women and ‘Arm LBM‐MAP’ in men. Adjustment by body fat or body fat percentage did not substantially modify the results (Tables S3 and S4). Body fat predominantly and positively associated with blood pressures in both sexes (Table S5).

## 4 Discussion

This study discloses the sex‐specific relationship of LBM with central haemodynamics and cardiac remodelling in a representative sample of the LBM spectrum throughout the adult lifespan. In both women and men, LBM strongly and positively associated with LV_mass_; however, LV structure progressed towards ‘hypertrophied’ concentric remodelling (LVRWTd) with higher LBM in men but not in women. Moreover, arterial blood pressure, specifically SBP, exclusively associated with LBM in men: The more the LBM, the higher the SBP. Discordant sex‐specific relationships were not observed in the peripheral circulation, in that LBM inversely associated with SVR in women and men.

The relationship of LBM with study variables seems to follow a continuum in the adult population, with women falling in the lower range and men in the upper range of the LBM spectrum (Figure 1). In this respect, the associations of LBM with SBP and LV concentric remodelling were manifest in the upper (men's) range of LBM (37–72 kg for total LBM). This approximately concurs with recent epidemiological findings denoting an inflection point (> 43 kg of LBM) after which the association of LBM with hypertension becomes positive [7]. Hence, the human body may have an absolute threshold for the amount of LBM that elicits detrimental central haemodynamic effects, predominantly comprising men—due to their intrinsically enhanced muscle mass partly attributed to their markedly higher androgen production relative to women. Consequently, LBM reduction in men with high LBM might enhance cardiac health. Small decreases in SBP (−10 mmHg) are independently associated with a 13% reduction in all‐cause mortality [8]. According to the current findings, a 12 kg curtailment of LBM in men associates with a 10 mmHg decrease in SBP (Figure 1), which might be achievable in individuals that have substantially increased their LBM via resistance training and/or anabolic drugs (e.g., bodybuilders). Nonetheless, the effect size of the association between LBM and SBP is small (LBM explains 10% of the variance in SBP in men), denoting the contribution of factors other than LBM.

In addition to the total amount of LBM, the body distribution of LBM is relevant, notably in women. Unlike total LBM, arm LBM is positively associated with SBP and LV concentric remodelling in women (Table S2). These arm‐specific associations were independent of body fat, contrasting with previous findings suggesting that adiposity explained the association of arm LBM with hypertension [9]. Although speculative, higher arm LBM, necessarily entailing increased arm blood flow, may result, paradoxically, in augmented SVR given the markedly reduced vascular conductance in the upper limb relative to other body regions [10]. Collectively considered, total LBM independently associates with a hypertensive CV phenotype in men, whereas regional LBM, specifically in the arms, is linked with the same detrimental phenotype in women. The analyses comprised healthy non‐obese adults to avoid the confounding impact of pathophysiological alterations; thus, they cannot yet be extrapolated to clinical and/or obese populations in which the present relationships might be altered or not present. Furthermore, the potential mediating role of sex hormones remains to be investigated in future studies.

## Conflicts of Interest

The authors declare no conflicts of interest.

## Supporting information


**Table S1:** Characteristics of study participants.
**Figure S1:** Frequency distribution of total and regional lean body mass (LBM) in women and men.
**Table S2:** Association of lean body mass (LBM) with peripheral/central haemodynamics, left ventricular mass (LVmass) and structure in women and men.
**Table S3:** Association of lean body mass (LBM) with peripheral/central haemodynamics, left ventricular mass (LVmass) and structure in women and men, adjusted by body fat^1^.
**Table S4:** Association of lean body mass (LBM) with peripheral/central haemodynamics, left ventricular mass (LVmass) and structure in women and men, adjusted by body fat percentage^1^.
**Table S5:** Association of body fat with peripheral/central haemodynamics, left ventricular mass (LVmass) and structure in women and men.

## Data Availability

All data associated with this study will be available upon reasonable request to the corresponding author.
