# Supplementary material for: Lean Body Mass Associates With a Hypertensive Cardiovascular Phenotype in Men but Not in Women
Source: J Cachexia Sarcopenia Muscle. 2025 Nov 10;16(6):e70125. doi: 10.1002/jcsm.70125 (PMC12598298; doi:10.1002/jcsm.70125)
Supplement: Supplementary file 1 — Table S1: Characteristics of study participants. Figure S1: Frequency distribution of total and regional lean body mass (LBM) in women and men. Table S2: Association of lean body mass (LBM) with peripheral/central haemodynamics, left ventricular mass (LVmass) and structure in women and men. Table S3: Association of lean body mass (LBM) with peripheral/central haemodynamics, left ventricular mass (LVmass) and structure in women and men, adjusted by body fat1. Table S4: Association of lean body mass (LBM) with peripheral/central haemodynamics, left ventricular mass (LVmass) and structure in women and men, adjusted by body fat percentage1. Table S5: Association of body fat with peripheral/central haemodynamics, left ventricular mass (LVmass) and structure in women and men. [file JCSM-16-e70125-s001.pdf]

SUPPLEMENTAL MATERIAL

- **Supplemental Table 1.** Characteristics of study participants.

- **Supplemental Figure 1.** Frequency distribution of total and regional lean body mass (LBM) in women and men.

- **Supplemental Table 2.** Association of lean body mass (LBM) with peripheral/central hemodynamics, left ventricular mass (LV_{mass}) and structure in women and men.

- **Supplemental Table 3.** Association of lean body mass (LBM) with peripheral/central hemodynamics, left ventricular mass (LV_{mass}) and structure in women and men, adjusted by body fat.

- **Supplemental Table 4.** Association of lean body mass (LBM) with peripheral/central hemodynamics, left ventricular mass (LV_{mass}) and structure in women and men, adjusted by body fat percentage.

- **Supplemental Table 5.** Association of body fat with peripheral/central hemodynamics, left ventricular mass (LV_{mass}) and structure in women and men.

Supplemental Table 1. Characteristics of study participants

	**Women**	**Men**	***P* value**
**General characteristics**			
n	162	163	—
Age (yrs)	42.9 ± 18.2	44.1 ± 18.5	0.595
Height (cm)	161.7 ± 6.8	174.1 ± 7.2	**<0.001**
Weight (kg)	56.9 ± 8.5	71.3 ± 11.2	**<0.001**
BMI (kg·m^{-2})	21.8 ± 2.6	23.5 ± 3.1	**<0.001**
BSA (m^2)	1.59 ± 0.13	1.85 ± 0.16	**<0.001**
SBP (mm Hg)	114.3 ± 20.0	124.4 ± 15.9	**<0.001**
DBP (mm Hg)	72.1 ± 13.2	75.6 ± 11.4	**0.012**
MVPA (hr·wk^{-1})	5.3 ± 3.4	5.9 ± 3.8	0.090
MVPA-END (hr·wk^{-1})	4.7 ± 3.3	5.2 ± 3.8	0.234
VO_{2peak} (ml·min^{-1}·kg^{-1})	32.3 ± 8.7	41.4 ± 11.2	**<0.001**
Smoking (%)	0	0	—
**Cardiac structure**			
LV_{mass} (g)	98.0 ± 29.4	142.4 ± 34.4	**<0.001**
IVSd (cm)	0.83 ± 0.18	0.98 ± 0.23	**<0.001**
LVIDd (cm)	3.98 ± 0.43	4.48 ± 0.49	**<0.001**
LVPWd (cm)	0.81 ± 0.19	0.91 ± 0.16	**<0.001**
LVWTd (cm)	1.65 ± 0.33	1.89 ± 0.34	**<0.001**
LVRWTd (cm)	0.42 ± 0.12	0.41 ± 0.09	0.697
**Body composition**			
Total BMC (kg)	1.9 ± 0.3	2.5 ± 0.4	**<0.001**
Total LBM (kg)	38.5 ± 5.3	54.4 ± 7.7	**<0.001**
Total body fat (kg)	16.6 ± 4.4	14.6 ± 4.8	**<0.001**
Total body fat %	28.9 ± 5.4	20.2 ± 4.8	**<0.001**
Leg LBM (kg)	12.8 ± 2.0	18.5 ± 2.8	**<0.001**
Leg fat (kg)	6.7 ± 1.7	5.0 ± 1.6	**<0.001**
Leg fat %	32.8 ± 5.8	20.1 ± 5.0	**<0.001**
Arm LBM (kg)	3.3 ± 0.8	5.9 ± 1.5	**<0.001**
Arm fat (kg)	1.9 ± 0.6	1.7 ± 0.6	**<0.001**
Arm fat %	35.0 ± 7.1	20.9 ± 5.6	**<0.001**
Trunk LBM (kg)	18.9 ± 2.8	25.8 ± 4.1	**<0.001**
Trunk fat (kg)	6.9 ± 2.3	6.6 ± 2.8	0.416
Trunk fat %	25.7 ± 6.6	19.5 ± 5.7	**<0.001**

Data are reported as mean ± SD.

Significant differences ($P < 0.05$) between women and men are highlighted in bold.

BMC, bone mineral content; BMI, body mass index; BSA, body surface area; DBP, diastolic blood pressure; IVSd, interventricular septum thickness at end-diatsole; LBM, lean body mass; LVIDd, left ventricular internal diameter at end-diastole; LV_{mass}, left ventricular mass; LVPWd, left ventricular posterior wall thickness at end-diastole; LVWTd, total left ventricular wall thickness at end-diastole; LVRWTd, left ventricular relative wall thickness at end-diastole; MVPA, total moderate-to-vigorous physical activity; MVPA-END, moderate-to-vigorous physical comprising endurance exercise; SBP, systolic blood pressure; VO_{2peak}, peak O_2 consumption.

Supplemental Figure 1. Frequency distribution of total and regional lean body mass (LBM) in women and men.

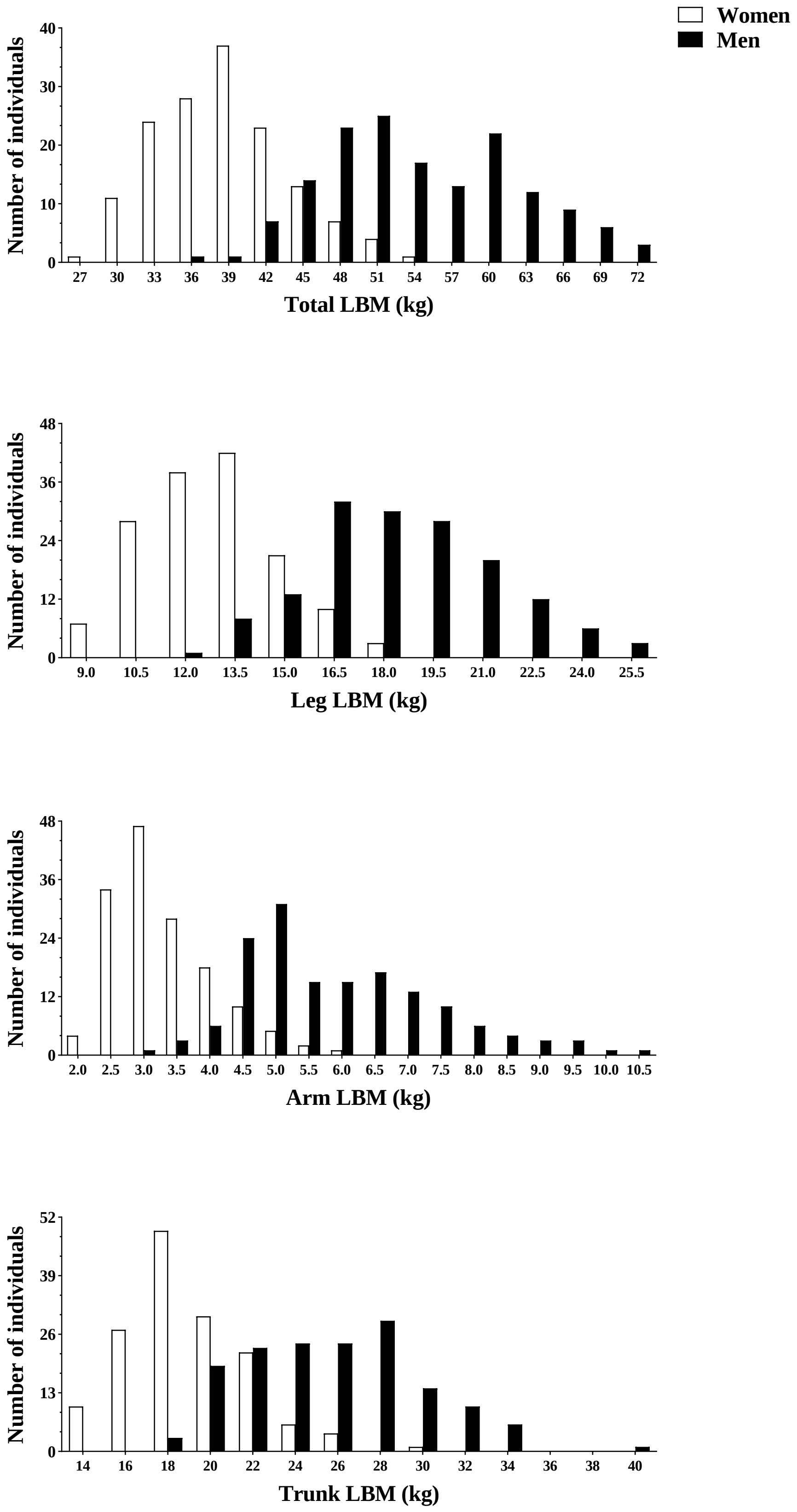

Each bilateral body region (leg LBM, arm LBM) is presented as the sum of LBM in right and left limbs.

Supplemental Table 2. Association of lean body mass (LBM) with peripheral/central hemodynamics, left ventricular mass (LV_{mass}) and structure in women and men

	**Total LBM (kg)**				**Leg LBM (kg)**				**Arm LBM (kg)**				**Trunk LBM (kg)**			
	**Women**		**Men**		**Women**		**Men**		**Women**		**Men**		**Women**		**Men**	
	r	***P***	***r***	***P***	***r***	***P***	***r***	***P***	***r***	***P***	***r***	***P***	***r***	***P***	***r***	***P***
SVR (dyn·s·cm^{-5})	-0.291	**<0.001**	-0.306	**<0.001**	-0.335	**<0.001**	-0.273	**<0.001**	-0.216	**0.008**	-0.256	**0.001**	-0.231	**0.005**	-0.317	**<0.001**
SBP (mm Hg)	0.094	0.256	0.307	**<0.001**	-0.044	0.592	0.262	**0.001**	0.296	**<0.001**	0.361	**<0.001**	0.175	**0.033**	0.325	**<0.001**
DBP (mm Hg)	0.037	0.651	0.028	0.733	-0.076	0.360	-0.002	0.979	0.169	**0.039**	0.066	0.420	0.111	0.176	0.029	0.718
MAP (mm Hg)	0.032	0.697	0.121	0.135	-0.103	0.213	0.096	0.239	0.178	**0.030**	0.164	**0.043**	0.130	0.115	0.132	0.103
LV_{mass} (g)	0.438	**<0.001**	0.537	**<0.001**	0.311	**<0.001**	0.455	**<0.001**	0.557	**<0.001**	0.458	**<0.001**	0.474	**<0.001**	0.551	**<0.001**
IVSd (cm)	0.219	**0.007**	0.390	**<0.001**	0.103	0.210	0.273	**<0.001**	0.416	**<0.001**	0.435	**<0.001**	0.276	**<0.001**	0.447	**<0.001**
LVIDd (cm)	0.297	**<0.001**	0.143	0.079	0.290	**<0.001**	0.205	**0.011**	0.161	**0.049**	-0.030	0.711	0.283	**<0.001**	0.085	0.295
LVPWd (cm)	0.246	**0.003**	0.384	**<0.001**	0.140	0.089	0.280	**<0.001**	0.412	**<0.001**	0.403	**<0.001**	0.272	**<0.001**	0.421	**<0.001**
LVWTd (cm)	0.258	**0.002**	0.450	**<0.001**	0.135	0.100	0.320	**<0.001**	0.458	**<0.001**	0.490	**<0.001**	0.303	**<0.001**	0.506	**<0.001**
LVRWTd (cm)	0.063	0.448	0.235	**0.003**	-0.020	0.806	0.125	0.124	0.250	**0.002**	0.331	**<0.001**	0.091	0.268	0.292	**<0.001**

Significant associations ($P<0.05$) are highlighted in bold.

All associations remained significant after adjustment by the family-wise error rate via the Benjamini-Hochberg procedure, except for ‘Arm LBM-DBP’, ‘Arm LBM-LVIDd’ and ‘Trunk LBM-SBP’ in women, and ‘Arm LBM-MAP’ in men ($P>0.05$).

DBP, brachial diastolic blood pressure; IVSd, interventricular septum thickness at end-diastole; LVIDd, left ventricular internal dimension at end-diastole; LVPWd, left ventricular posterior wall thickness at end-diastole; LVRWTd, left ventricular relative wall thickness at end-diastole; LVWTd, total left ventricular wall thickness at end-diastole; MAP, brachial mean arterial pressure; *r*, Pearson correlation coefficient; SBP, brachial systolic blood pressure; SVR, systemic vascular resistance.

Supplemental Table 3. Association of lean body mass (LBM) with peripheral/central hemodynamics, left ventricular mass (LV_{mass}) and structure in women and men, adjusted by body fat[1]

	**Total LBM (kg)**				**Leg LBM (kg)**				**Arm LBM (kg)**				**Trunk LBM (kg)**			
	**Women**		**Men**		**Women**		**Men**		**Women**		**Men**		**Women**		**Men**	
	r	***P***	***r***	***P***	***r***	***P***	***r***	***P***	***r***	***P***	***r***	***P***	***r***	***P***	***r***	***P***
SVR (dyn·s·cm^{-5})	-0.301	**<0.001**	-0.287	**<0.001**	-0.319	**<0.001**	-0.251	**0.002**	-0.208	**0.011**	-0.235	**0.004**	-0.278	**<0.001**	-0.315	**<0.001**
SBP (mm Hg)	0.048	0.562	0.243	**0.003**	-0.062	0.451	0.244	**0.002**	0.235	**0.004**	0.290	**<0.001**	0.108	0.191	0.201	**0.013**
DBP (mm Hg)	-0.006	0.939	-0.046	0.572	-0.089	0.283	-0.021	0.798	0.123	0.138	0.001	0.994	0.041	0.621	-0.119	0.146
MAP (mm Hg)	-0.024	0.773	0.032	0.694	-0.119	0.151	0.073	0.373	0.115	0.163	0.080	0.325	0.041	0.623	-0.047	0.563
LV_{mass} (g)	0.434	**<0.001**	0.535	**<0.001**	0.301	**<0.001**	0.461	**<0.001**	0.527	**<0.001**	0.437	**<0.001**	0.484	**<0.001**	0.544	**<0.001**
IVSd (cm)	0.222	**0.007**	0.369	**<0.001**	0.102	0.219	0.289	**<0.001**	0.388	**<0.001**	0.392	**<0.001**	0.285	**<0.001**	0.384	**<0.001**
LVIDd (cm)	0.286	**<0.001**	0.142	0.082	0.280	**<0.001**	0.181	**0.026**	0.150	0.070	-0.015	0.853	0.277	**<0.001**	0.127	0.120
LVPWd (cm)	0.246	**0.003**	0.404	**<0.001**	0.131	0.112	0.312	**<0.001**	0.380	**<0.001**	0.397	**<0.001**	0.287	**<0.001**	0.428	**<0.001**
LVWTd(cm)	0.259	**0.001**	0.445	**<0.001**	0.129	0.117	0.347	**<0.001**	0.427	**<0.001**	0.459	**<0.001**	0.316	**<0.001**	0.469	**<0.001**
LVRWTd (cm)	0.061	0.461	0.249	**0.002**	-0.026	0.750	0.162	**0.046**	0.217	**0.008**	0.317	**<0.001**	0.098	0.238	0.275	**<0.001**

Significant associations (*P*<0.05) are highlighted in bold.

[1] All associations were adjusted by total or regional-specific body fat, as applicable.

DBP, brachial diastolic blood pressure; IVSd, interventricular septum thickness at end-diastole; LVIDd, left ventricular internal dimension at end-diastole; LVPWd, left ventricular posterior wall thickness at end-diastole; LVRWTd, left ventricular relative wall thickness at end-diastole; LVWTd, total left ventricular wall thickness at end-diastole; MAP, brachial mean arterial pressure; *r*, Pearson correlation coefficient; SBP, brachial systolic blood pressure; SVR, systemic vascular resistance.

Supplemental Table 4. Association of lean body mass (LBM) with peripheral/central hemodynamics, left ventricular mass (LV_{mass}) and structure in women and men, adjusted by body fat percentage[1]

	**Total LBM (kg)**				**Leg LBM (kg)**				**Arm LBM (kg)**				**Trunk LBM (kg)**			
	**Women**		**Men**		**Women**		**Men**		**Women**		**Men**		**Women**		**Men**	
	r	***P***	***r***	***P***	***r***	***P***	***r***	***P***	***r***	***P***	***r***	***P***	***r***	***P***	***r***	***P***
SVR (dyn·s·cm^{-5})	-0.257	**0.002**	-0.306	**<0.001**	-0.329	**<0.001**	-0.297	**<0.001**	-0.184	**0.025**	-0.255	**0.002**	-0.207	**0.012**	-0.321	**<0.001**
SBP (mm Hg)	0.149	0.070	0.336	**<0.001**	0.006	0.945	0.274	**<0.001**	0.383	**<0.001**	0.411	**<0.001**	0.209	**0.011**	0.320	**<0.001**
DBP (mm Hg)	0.092	0.266	0.062	0.451	-0.031	0.710	0.018	0.826	0.236	**0.004**	0.127	0.118	0.146	0.076	0.008	0.922
MAP (mm Hg)	0.104	0.208	0.166	**0.041**	-0.050	0.549	0.118	0.147	0.280	**<0.001**	0.241	**0.003**	0.178	**0.030**	0.115	0.158
LV_{mass} (g)	0.419	**<0.001**	0.529	**<0.001**	0.301	**<0.001**	0.427	**<0.001**	0.559	**<0.001**	0.439	**<0.001**	0.462	**<0.001**	0.555	**<0.001**
IVSd (cm)	0.203	**0.013**	0.393	**<0.001**	0.097	0.240	0.243	**0.003**	0.411	**<0.001**	0.444	**<0.001**	0.266	**0.001**	0.442	**<0.001**
LVIDd (cm)	0.289	**<0.001**	0.137	0.093	0.276	**<0.001**	0.224	**0.005**	0.158	0.056	-0.046	0.576	0.279	**<0.001**	0.095	0.246
LVPWd (cm)	0.233	**0.004**	0.371	**<0.001**	0.150	0.068	0.230	**0.004**	0.423	**<0.001**	0.375	**<0.001**	0.261	**0.001**	0.425	**<0.001**
LVWTd(cm)	0.242	**0.003**	0.444	**<0.001**	0.138	0.094	0.278	**<0.001**	0.462	**<0.001**	0.482	**<0.001**	0.292	**<0.001**	0.504	**<0.001**
LVRWTd (cm)	0.066	0.426	0.529	**0.005**	0.005	0.955	0.075	0.361	0.278	**<0.001**	0.317	**<0.001**	0.088	0.287	0.291	**<0.001**

Significant associations (P<0.05) are highlighted in bold.

[1] All associations were adjusted by total or regional-specific body fat percentage, as applicable.

DBP, brachial diastolic blood pressure; IVSd, interventricular septum thickness at end-diastole; LVIDd, left ventricular internal dimension at end-diastole; LVPWd, left ventricular posterior wall thickness at end-diastole; LVRWTd, left ventricular relative wall thickness at end-diastole; LVWTd, total left ventricular wall thickness at end-diastole; MAP, brachial mean arterial pressure; *r*, Pearson correlation coefficient; SBP, brachial systolic blood pressure; SVR, systemic vascular resistance.

Supplemental Table 5. Association of body fat with peripheral/central hemodynamics, left ventricular mass (LV_{mass}) and structure in women and men

	**Total Fat (kg)**				**Leg Fat (kg)**				**Arm Fat (kg)**				**Trunk Fat (kg)**			
	**Women**		**Men**		**Women**		**Men**		**Women**		**Men**		**Women**		**Men**	
	r	***P***	***r***	***P***	***r***	***P***	***r***	***P***	***r***	***P***	***r***	***P***	***r***	***P***	***r***	***P***
SVR (dyn·s·cm^{-5})	0.009	0.914	-0.113	0.163	-0.131	0.112	-0.155	0.055	-0.060	0.468	-0.108	0.182	0.117	0.157	-0.078	0.336
SBP (mm Hg)	0.206	**0.012**	0.278	**<0.001**	0.092	0.262	0.131	0.106	0.313	**<0.001**	0.308	**<0.001**	0.258	**0.002**	0.358	**<0.001**
DBP (mm Hg)	0.187	**0.022**	0.231	**0.004**	0.064	0.440	0.098	0.229	0.206	**0.012**	0.199	**0.014**	0.254	**0.002**	0.302	**<0.001**
MAP (mm Hg)	0.236	**0.004**	0.302	**<0.001**	0.076	0.355	0.133	0.102	0.276	**<0.001**	0.279	**<0.001**	0.320	**<0.001**	0.394	**<0.001**
LV_{mass} (g)	0.071	0.393	0.101	0.215	0.088	0.284	0.011	0.897	0.252	**0.002**	0.153	0.059	0.038	0.643	0.156	0.055
IVSd (cm)	0.014	0.867	0.138	0.089	0.018	0.823	-0.055	0.500	0.183	**0.026**	0.228	**0.005**	0.012	0.888	0.258	**<0.001**
LVIDd (cm)	0.084	0.306	0.025	0.757	0.085	0.300	0.158	0.051	0.066	0.424	-0.049	0.551	0.065	0.433	-0.066	0.421
LVPWd (cm)	0.029	0.726	-0.002	0.982	0.061	0.458	-0.130	0.110	0.205	**0.012**	0.091	0.265	-0.007	0.929	0.085	0.297
LVWTd(cm)	0.024	0.772	0.092	0.259	0.045	0.586	-0.100	0.218	0.215	**0.008**	0.198	**0.014**	0.002	0.981	0.215	**0.008**
LVRWTd (cm)	0.014	0.864	-0.006	0.939	0.031	0.707	-0.169	**0.037**	0.166	**0.043**	0.102	0.208	-0.007	0.930	0.104	0.199

Significant associations ($P<0.05$) are highlighted in bold.

DBP, brachial diastolic blood pressure; IVSd, interventricular septum thickness at end-diastole; LVIDd, left ventricular internal dimension at end-diastole; LVPWd, left ventricular posterior wall thickness at end-diastole; LVRWTd, left ventricular relative wall thickness at end-diastole; LVWTd, total left ventricular wall thickness at end-diastole; MAP, brachial mean arterial pressure; *r*, Pearson correlation coefficient; SBP, brachial systolic blood pressure; SVR, systemic vascular resistance.
